# Melanoma Arising in Tattoos: A Case Series and Scoping Review of the Literature

**DOI:** 10.3390/cancers17050767

**Published:** 2025-02-24

**Authors:** Marco Brusasco, Sofia Spagnolini, Laura Mazzoni, Serena Magi, Giuseppe Scarcella, Ignazio Stanganelli

**Affiliations:** 1 Dermatology Unit, ASST Santi Paolo e Carlo, 20142 Milano, Italy; marco.brusasco@asst-santipaolocarlo.it; 2 Dermatology Resident Training Program, Department of Medicine and Surgery, University of Parma, 43121 Parma, Italy; ignazio.stanganelli@irst.emr.it; 3 Skin Cancer Unit, IRCCS Istituto Romagnolo per lo Studio dei Tumori “Dino Amadori”—IRST, 47014 Meldola, Italy; laura.mazzoni@irst.emr.it (L.M.); serena.magi@irst.emr.it (S.M.); 4 Dermatology Unit, Hospital G. Fracastoro San Bonifacio, 37047 Verona, Italy; gs@giuseppescarcella.it

**Keywords:** melanoma, skin neoplasm, tattoo, dermoscopy, confocal microscopy

## Abstract

Background/Objectives: The prevalence of tattoos has risen globally in recent decades, ranging from 10% to 29%. Although rare, melanomas arising within tattoos are challenging for dermatologists due to the complexities in clinical and dermoscopic evaluation. In this article, we report two cases of melanoma on tattoos, review the reported cases in the literature, and examine the role of reflectance confocal microscopy (RCM) in improving the diagnosis of melanoma on tattooed skin. Methods: We conducted a systematic literature search on Medline/Pubmed for the period from the inception of the databases to 31 October 2024, using the Mesh major topics ‘melanoma’ AND ‘tattoo’ OR ‘tattoo skin tumor’. Out of the 268 citations identified by our search, 37 studies met the eligibility criteria. Results: In total, 43 cases of melanoma arising on tattooed skin were identified, to which we add our 2 cases, bringing the total to 45. The most common locations were the upper limbs (53%) and trunk (38%), predominantly arisen on black and blue tattoos. Of 40 cases with known depth of invasion, 4 were in situ and 36 invasive, with a mean Breslow thickness of 2.7 mm. Seven patients had a positive sentinel lymph node. Of 16 cases with an available horizontal diameter, 12 melanomas had a diameter of ≥1 cm. Conclusions: The limited cases of ‘’melanoma on tattoos” reported in the literature suggest a coincidental association rather than a direct causal link. Nevertheless, increased awareness among patients and tattoo artists about potential risks and preventive measures may enhance the management of melanocytic lesions in tattooed individuals. Lastly, integrating reflectance confocal microscopy with dermoscopy increases the overall diagnostic accuracy for melanoma, enhancing the identification of pigmented and non-pigmented skin lesions.

## Simple Summary

The prevalence of tattoos has risen globally in recent decades, ranging from 10% to 29%. While the carcinogenic risk of tattoo inks is still debated, their confounding effect in assessing melanocytic lesions is well-documented. Melanomas arising within tattoos are rare but challenging for dermatologists due to the diagnostic complexities in clinical and dermoscopic evaluation. This article reviews cases of melanoma on tattooed skin in the literature, introduces two new cases, and examines the potential role of reflectance confocal microscopy combined with dermoscopy in improving diagnostic accuracy for melanoma and the decision-making process in the setting of flat melanocytic skin lesions on tattooed areas.

## 1. Introduction

The percentage of the tattooed population has steadily increased in recent decades, with tattoo prevalence ranging between 10% and 29% [1].

Despite limited evidence, several factors have been proposed as playing a pathogenic role in the development of malignant tumors on tattooed skin: local trauma from ink injection; inflammatory response triggered by ink; ink composition and its metabolites; ink photoreactive properties [2,3].

Tattoo inks have a complex formulation, including organic colorants and various metal salts. Certain ingredients, such as polycyclic aromatic hydrocarbons (PAHs), nitrosamines, and metal salts, are known for their carcinogenic properties [4,5,6,7]. Moreover, additional potentially toxic substances may be formed by the degradation of inks through UV radiation and the laser removal of tattoos. It has also been demonstrated that tattoo pigments are present in tissues other than the skin and can be metabolized by human enzymes [8].

While the carcinogenic potential of tattoo inks remains a topic of debate, their confounding effect in assessing pigmented lesions, particularly melanocytic nevi, is well-documented. Although cases of melanoma arising within tattoos are rare, they present unique diagnostic and management challenges for dermatologists due to the difficulties in clinical and dermoscopic evaluation.

In this article, we analyze all the reported cases in the literature of melanoma arising in tattooed skin, and present two new cases from our clinic, examining the potential role of reflectance confocal microscopy (RCM) in improving diagnostic accuracy for melanomas and the decision-making process in the management of melanocytic skin lesions on tattooed areas [9,10].

## 2. Case Series

### 2.1. Case 1

**Figure 1:**
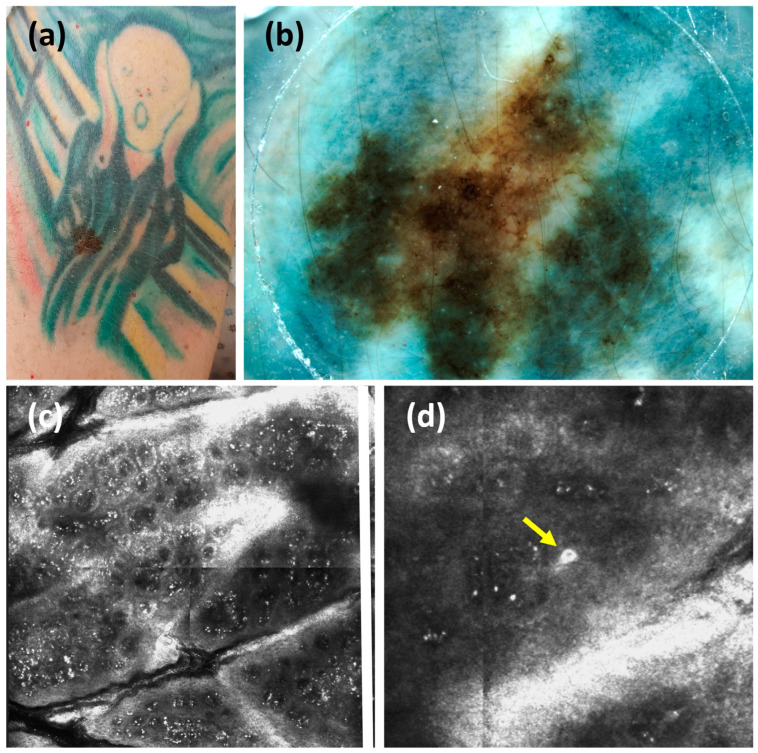
(**a**) Case 1: a clinical suspicious melanocytic lesion based on ABCD rules, arisen in a multi-colored tattoo depicting Munch’s painting “The Scream”. (**b**) The dermoscopy of the lesion is hindered by dark blue-green ink pigments (20×). (**c**,**d**) RCM evaluation showed ring and edge papillae with irregular, hyper-refractile tattoo particles, and the presence of roundish cells with a central nucleus (yellow arrow) in the spinous layer ((**c**): 1 × 1 mm; (**d**): 0.7 × 0.7 mm).

A 47-year-old man, previously followed at another clinic, presented for routine mole screening. The patient had atypical mole syndrome, a positive family history of melanoma (maternal aunt), and had a personal history of multiple melanomas, specifically an invasive melanoma pT1b, with a negative sentinel lymph node four years prior and a melanoma in situ two years prior. During the examination, on the right arm was noted an atypical mole covered by the ink of a multi-colored tattoo depicting Munch’s “The Scream”. Even if clinically suspicious under the ABCD rule (Figure 1a), the lesion’s dermoscopic evaluation was hindered by the dark blue-green ink (Figure 1b). The patient was hesitant about the surgical excision, as the procedure would have damaged a tattoo with high personal significance. Moreover, we performed confocal microscopy to provide additional cytological and architectural RCM features, including the presence of reflecting roundish cells irregularly distributed in the spinous layer, histologically related to pagetoid melanocytosis [10] (Figure 1c,d). The histological analysis, following the excision, revealed a superficial spreading melanoma with a Breslow thickness of 0.4 mm, no ulceration, regression features < 75%, and 1 mitosis per mm^2^. Abdominal and total body lymph node ultrasound were negative for oncological findings. The final staging was pT1a, according to the VIII edition of the American Joint Committee on Cancer (AJCC) staging system.

### 2.2. Case 2

**Figure 2:**
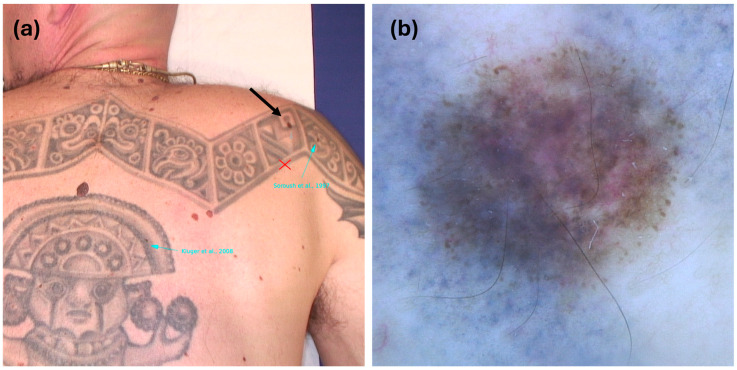
(**a**) Case 2: black arrow pointing at clinical suspicious melanocytic lesion on black tribal tattoo. (**b**) Dermoscopy showed peripheral atypical globules, atypical pigment network, and milky red areas in center of lesion (30×).

A 40-year-old man with a personal history of in situ melanoma excised six years earlier, no family history of melanoma, and diffuse dysplastic nevi presented for a follow-up mole screening. During the examination, an atypical mole was noted on the upper back, partially covered by a black tattoo. The dermoscopic evaluation revealed peripheral atypical globules, reticular disorganization, and milky red areas in the center of the lesion, raising suspicion for melanoma (Figure 2). The RCM of the lesion was not performed as it was unavailable in the clinic at the time of the visit. The histological analysis, following the excision, confirmed a superficial spreading melanoma with a Breslow thickness of 0.4 mm, no ulceration, regression features < 75%, and 0 mitoses per mm^2^. Abdominal and total body lymph node ultrasound showed no oncological findings. The final staging was pT1a, according to the VIII edition of the AJCC staging system.

## 3. Materials and Methods

**Figure 3:**
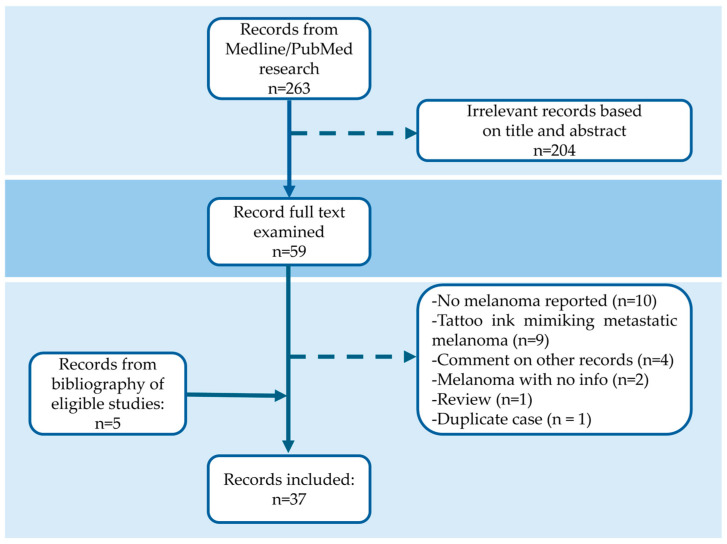
Flow chart of literature review.

We searched for original primary articles on patients with melanoma arising within a tattoo. We conducted a systematic literature search on Medline/Pubmed for the period from the inception of the databases to 31 October 2024, using the Mesh major topics ‘melanoma’ AND ‘tattoo’ OR ‘tattoo skin tumor’. Reference lists of eligible studies were also reviewed to identify additional publications. All article types were included if the full text was available and reporting one or more cases of melanoma arising within a tattoo. From the identified cases, we analyzed the following characteristics: patient demographics, tattoo color in the affected area, melanoma features (body site, horizontal diameter, Breslow thickness, sentinel lymph node biopsy), the duration of the tattoo prior to consultation, time interval between tattoo acquisition and lesion appearance, time interval between lesion onset and diagnosis, family history of melanoma, history of previous cancers—including melanoma and non-melanoma skin cancer (NMSC)—and the history of chronic sun exposure. Out of the 268 citations identified by our search, 37 studies met the eligibility criteria (Figure 3).

## 4. Results

In total, 43 cases of melanoma arising on tattooed skin were identified, to which we add our 2cases, bringing the total to 45 (40 males, 4 females, 1 not specified), with a median age of 45.7 years (Table 1). The most common locations were the upper limbs (53%) and trunk (38%). Melanomas predominantly arose on black or blue tattoos (34 out of 37 cases reporting color). A pre-existing mole at the tattoo site was reported by eight patients. Family history of melanoma was available for nine cases and only one patient had a positive family history. Among the 16 cases where a personal history of tumors was reported, 5 patients had previous skin cancers. Specifically, there were two cases of melanoma, one case with two melanomas, one case of basal cell carcinoma, and one case with both a melanoma and a non-melanoma skin cancer. Moreover, 13 patients (29%) reported chronic sun exposure. In 40 out of 45 cases, the depth of invasion was known: 36 were invasive melanomas and only 4 were in situ, with a median Breslow thickness of 2.7 mm. Seven patients had a positive sentinel lymph node. Of the 16 melanomas with an available horizontal diameter data, 15 had a diameter > 6 mm and 12 cases had a diameter ≥ 1 cm.

## 5. Discussion

Over the past few decades, the prevalence of tattooed individuals has increased to a rate ranging between 10% and 29% worldwide [1]. Consequently, there has been a rise in reports of adverse events, including infections, allergic reactions, and both benign and malignant tumors. Although rare, reported cutaneous malignancies on tattooed skin include malignant melanoma, squamous cell carcinoma, basal cell carcinoma, keratoacanthoma, dermatofibrosarcoma protuberans, and other tumors [40]. Despite limited evidence, several factors have been proposed as playing a pathogenic role in the development of malignant tumors on tattooed skin: local trauma from ink injection; inflammatory response triggered by ink; ink composition and its metabolites; and ink photoreactive properties [2,3].

Since January 2022, in Europe, tattoo and permanent make-up inks have been regulated under the Registration, Evaluation, Authorisation and Regulation of Chemicals (REACH) framework, which provides concentration limits for hazardous chemicals in consumer products [46]. This new regulation led to the banning or restriction of more than 4000 chemical substances under Entry 75 of Annex XVII of Regulation (EC) No. 1907/2006. Nevertheless, it remains unclear to what extent tattoo ink manufacturers will be able to produce compliant inks of sufficient quality for artistic tattoo purposes [47].

Tattoo inks are complex mixtures of inorganic (mostly industrial dyes) and organic pigments (e.g., azo pigments), suspended in solvents like water or alcohol, along with additional components such as preservatives, thickeners, or binders (Table 2). These pigments are often not manufactured specifically for tattooing, but for industrial purposes, meaning their purity may not meet the standards expected for safe cosmetic use. Notably, some of the substances used in tattoo inks have been classified by the International Agency for Research on Cancer (IARC) as carcinogenic (Group 1), probably carcinogenic (Group 2A), or possibly carcinogenic (Group 2B) to humans [4,8].

Commercially analyzed inks revealed various heavy metals, both as impurities and as pigment, including chromium (green and blue colorants), cobalt (yellow and blue colorants), lead, antimony, arsenic, and beryllium, as well as nickel and mercury (red colorant). Moreover, the presence of aromatic amines, phthalates, polycyclic aromatic hydrocarbons, and nanoparticles has been confirmed [5,7]. Different substances, especially azo pigments, have been proved to release potentially carcinogenic aromatic amines when exposed to solar, UV, or laser irradiation [8]. It has also been demonstrated that tattoo pigments are present in tissues other than skin, such as lymph nodes and Kupffer cells, and can be metabolized by human enzymes [8].

Black ink, the most commonly used color, contains significant levels of PAHs [48], and a prolonged exposure to these compounds has been associated with an increased risk of melanoma development [49]. Interestingly, reviews have observed that melanoma and basal cell carcinomas appear more frequently on darkly pigmented tattoos, whereas squamous cell carcinomas and keratoacanthomas are more commonly found on red tattoos, suggesting that different ink pigments may have distinct carcinogenic effects [3,50].

Despite the intriguing hypotheses linking tattoos and melanoma, the overall impact of tattoo ink on long-term skin health remains unclear, and the relatively few cases of tattoo-related skin cancers in the literature suggest that this association may be coincidental, rather than indicative of a direct causal link.

If the carcinogenic role of tattoo ink remains uncertain, tattoos clearly pose diagnostic and management challenges for melanocytic lesions. Tattoo ink, especially darker pigments, may alter the clinical and dermoscopic evaluation of melanocytic lesions, as seen in our two cases: in Case 2, the lesion was only partially covered by ink, and the fainted color allowed for the visualization of peripheral globules, regression areas, and a fragmented reticular pattern. In Case 1, however, the dark blue-green pigment did not completely cover the lesion but played a role in rendering the dermoscopic assessment more difficult.

This confounding effect can delay the diagnosis and potentially worsen patient outcomes. Moreover, tattoo pigments may interfere with the interpretation of sentinel lymph nodes, as pigment-laden macrophages can be mistaken for metastatic melanoma [51]. Melanomas arising on tattoos often have a deep Breslow thickness and are at a high risk of progression: our literature review identified only 4 cases of melanoma in situ, while 36 were invasive, of which 11 had a Breslow thickness > 2 mm. Furthermore, 7 of the 12 cases with known sentinel lymph node status were positive. For these advanced clinical–pathological stages, melanomas appearing on tattoos have negative prognostic features.

In view of these considerations, an active education held by dermatologists addressing both tattoo artists and patients is essential. Tattoos, although not contraindicated, should be discouraged in patients with a history of melanoma, particularly over surgical scars. A thorough evaluation of nevi prior to having a tattoo is strongly recommended for patients with a history of melanoma or risk factors for melanoma (such as family history, a high number of nevi, or atypical mole syndrome). It is important to maintain a free margin around nevi, and large à-plat coloring should be discouraged [52]. Among the cases of melanoma on tattoos, family history of melanoma and personal history of tumors were not well documented. In fact, data were available in only 9 cases for family history of melanoma and in 16 cases for previous tumors, of which 5 patients had previous skin cancers. We acknowledge their potential relevance and recommend that future case reports provide more detailed information on these variables to enhance clinical understanding of melanoma progression.

Professional tattoo artists are generally aware that they should not tattoo over moles and typically inspect the skin of their clients before they start tattooing [53]. Unfortunately, errors may occur, as for the patient in Case 1, or nevi and melanomas may also develop after the tattoo has been applied.

It has also been suggested that a screening at time 0 (before tattoo), this by archiving iconographic documentation of the skin, and more tightening follow up procedures should be attempted, especially in cases of very wide tattoos [42]; however, we align with Kluger’s opinion that since tattooing is not a real risk factor for melanoma, there is no justification for recommending ‘tightened’ surveillance for any tattooed individual, considering that almost 20% of the adult population is tattooed [54]. In our opinion and based on personal experience, a strict follow-up and photographic documentation (such as total body photography and digital dermoscopy) should be reserved for tattooed patients with a high number of nevi and an increased risk of melanoma.

Diagnosis of skin lesions appearing on the tattoo area can also be challenging for experienced dermatologists, since dermoscopic patterns can be overlapping or hidden by tattoos. In this difficult diagnostic context, the addition of reflectance confocal microscopy provides a valuable contribution, including the identification of benign or malignant patterns of melanocytic and non-melanocytic skin lesions [9,10,55,56,57,58]. RCM provides in vivo horizontal virtual skin sections of the skin, at a quasi-histological resolution, capturing details from the epidermal surface down to the upper dermis and allowing dynamic observation of cytological and structural skin changes. Several studies have shown that different skin pigmentation disorders display characteristic patterns on confocal imaging, including exogenous pigmentations [9,10,55,56,57,58].

**Figure 4:**
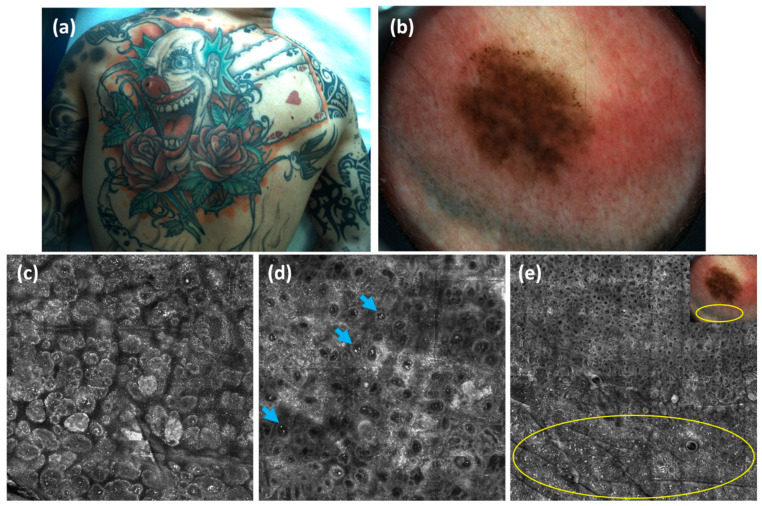
(**a**) A 29-year-old male from our clinic had a roundish dark brown macula located in the red area of the nose in a giant clown tattoo that completely covered the patient’s back. (**b**) Dermoscopic examination showed irregular diffuse pigmentation in the center with irregularly distributed brown globules at the periphery (20×). (**c**–**e**) RCM revealed dense nests at the dermal–epidermal junction with cytologic atypia, melanophages (blue arrow), associated with irregular, hyper-refractive tattoo particles (yellow circle). Due to the presence of atypical dermoscopic and RCM features, a biopsy was recommended. Histological evaluation showed a dysplastic nevus ((**c**,**d**): 0.5 × 0.5 mm; (**e**): 5 × 5 mm).

**Figure 5:**
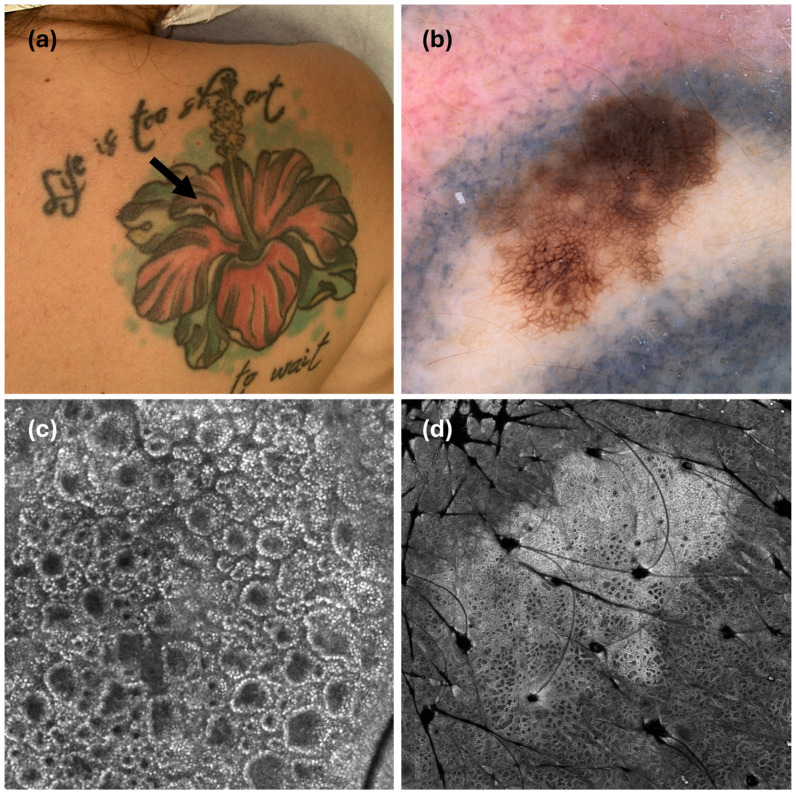
(**a**) A 33-year-old woman from our clinic exhibited a brown asymmetric macula (black arrow) located in the central area in a large multicolor tattoo on the right shoulder blade. (**b**) Dermoscopic analysis showed diffuse irregular pigmentation, a whitish veil, atypical pigment network, and numerous pigment dots irregularly distributed in the area hidden by the blue color of the tattoo (20×). (**c**,**d**) RCM revealed a typical pattern with regular rings and edge papillae at the dermal–epidermal junction, closely related to a melanocytic nevus ((**c**): 0.5 × 0.5 mm; (**d**): 5 × 5 mm).

O’goshi et al. described RCM features of normal skin containing tattoo ink, highlighting differences among ink colors: dark pigments were more often aggregated into large clusters in the superficial dermis, while colored inks appeared more diffusely distributed [56]. In another study, Reilly et al. retrospectively analyzed RCM features in 19 benign melanocytic lesions on tattooed skin. In all cases, evaluators were able to clearly distinguish irregular, hyper-refractile tattoo particles from melanocytic cells, allowing for accurate diagnosis [57]. In cases of equivocal melanocytic lesions, combining RCM and dermoscopy can reduce unnecessary excisions and enhance the accuracy of clinical management decisions, ultimately improving patient outcome patterns (Figure 4 and Figure 5).

Furthermore, RCM can be used to analyze non-melanocytic skin lesions such as basal cell carcinoma, early squamous cell carcinoma, and seborrheic keratosis/solar lentigo/lichen planus-like keratosis [9,58].

Among the case reports of melanoma arising on tattooed skin, only one study [2] included a confocal examination, which showed moderate atypia at the dermo-epidermal junction. For the patient in Case 1, the dermoscopic analysis of the melanoma within the tattoo was difficult because of the blue-green pigment. The patient was also hesitant about its removal, as the procedure would have damaged a highly significant personal tattoo. However, RCM proved crucial, revealing features strongly suggestive of malignancy.

Despite the limited available literature, our case highlights the potential utility of RCM in assessing melanocytic lesions covered by tattoos, thereby overcoming the limitations of dermoscopy in this specific subset of patients. Therefore, an increase in studies in this field is desirable to confirm this hypothesis.

## 6. Conclusions

The relatively few cases of melanoma arising on tattoos in the literature suggest that any potential association may be coincidental rather than indicative of a direct causal link. Although some findings point to a possible carcinogenic role of tattoo ink, current evidence remains insufficient to confirm this hypothesis.

Although the risk of developing melanoma on tattooed skin appears low, increased awareness among patients and tattoo artists about potential risks and preventive measures may enhance the management of melanocytic lesions in tattooed individuals.

Additionally, a close collaboration between dermatologists and tattoo artists, coupled with the use of advanced diagnostic tools like the RCM integrated with dermoscopy, could improve early melanoma detection and reduce the risk of delayed melanoma diagnoses.

Lastly, RCM shows promise in distinguishing melanocytic structures from exogenous pigment and provides additional diagnostic information useful in the decision-making process. However, further research is needed to confirm the efficacy of RCM in tattooed patients.

## Figures and Tables

**Table 1:** Cases of melanoma arising on tattoos, from 1938 to present date. BCC: Basal Cell Carcinoma; MM: Malignant Melanoma; NA: Not Available; NMSC: Non-Melanoma Skin Cancer.

Author	Sex	Age (Years)	Duration of Tattoo Prior to Consultation (Years)	Period from Acquiring Tattoo to Onset of Lesion (Years)	Period from Onset of Lesion to Diagnosis (Years)	Site	Family History of Melanoma	Previous Cancers	Chronic Sun Exposure	Decorative	Color of Affected Area	Breslow (mm)	SLNB	Size (mm)
Sharlit, 1938 [11]	M	9	3 months	NA	NA	Forehead	NA	NA	NA	No	Indelible mark caused by pencil puncture	NA	NA	NA
Allen, 1954 [12]	M	NA	NA	NA	NA	Arm	NA	NA	NA	NA	NA	NA	NA	NA
Kirsch, 1972 [13]	M	52	27	26	1	Right arm	NA	NA	NA	Yes	Blue	NA	Positive	NA
Wolford et al., 1974 [14]	M	55	29	24	5	Right arm	NA	NA	NA	Yes	Red	NA	NA	NA
Bartal et al., 1980 [15]	F	52	26	2	NA	Chest	NA	Intraductal breast adenocardinoma	NA	No: radiation field for breast tumor	Black (Indian ink)	Clark II	NA	5 × 10
	M	34	6	6	NA	Left forearm	NA	Hodgkin’s disease stage IIB	NA	No: radiation field for Hodgkin lymphoma	Black (Indian ink)	Clark IIB	NA	NA
Lee and Craig, 1984 [16]	M	44	>20	>16	4	Chest	NA	No	NA	Yes	Blue	2.5	NA	NA
Kircik et al., 1993 [17]	F	36	10	9	1	Right scapula	NA	NA	NA	Yes	Dark blue/green	1.1	NA	NA
Soroush et al., 1997 [18]	M	47	20	16	4	Abdomen	NA	NA	Yes	Yes	Black	0.7	NA	NA
Khan et al., 1999 [19]	M	44	25	NA	NA	Right forearm	No	No	Yes	Yes	Black/blue	0.9	NA	NA
Stinco et al., 2003 [20]	M	26	NA	Pre-existing nevus	Change in appearance over 3 years	Left scapula	NA	NA	NA	Yes	NA	0.9	Negative	15 × 25
Paradisi et al., 2006 [21]	M	36	10	Pre-existing nevus	Change in appearance over 1 year	Left scapula	No	No	Yes (no sunburn)	Yes	Dark blue	0.3	No	10 × 15
Shariff et al., 2006 [22]	M	48	30	30	3 moths	Right arm	NA	NA	NA	NA	NA	NA	NA	NA
Singh et al., 2007 [23]	M	56	NA	NA	NA	Right forearm	NA	NA	NA	Yes	NA	11	Positive + pigment tattoo	NA
Kluger et al., 2008 [24]	M	70	>40	NA	NA	Left upper arm	NA	NA	Yes	Yes	Dark blue	8	NA	15 × 15
Jaigirdar et al., 2009 [25]	M	64	NA	NA	NA	Right deltoid	NA	No	NA	Yes	NA	1.2	Positive + pigment tattoo	NA
	M	29	NA	NA	NA	Left forearm	NA	No	NA	Yes	NA	3.9	Positive + pigment tattoo	NA
Varga et al., 2011 [26]	M	28	5	Pre-existing nevus (4 years)	Change in appearance over 1 year	Left upper arm	NA	NA	NA	Yes	Black	1	Negative + pigment tattoo	13 × 15
Nolan et al., 2013 [27]	M	79	>60	NA	NA	Left arm	NA	NA	NA	Yes	Dark blue	0.2	No	NA
Körner et al., 2013 [28]	M	45	NA	NA	NA	Left lower leg	NA	NA	NA	Yes	Black	7	NA	NA
Pohl et al., 2013 [29]	M	29	10	NA	7	Right arm	NA	NA	NA	Yes	Black	0.4	No	NA
Kluger et al., 2014 [30]	M	61	NA	No	NA	Right thigh	NA	NA	NA	Yes	Black	15	Negative	NA
	M	32	2	NA	5	Upper back	NA	NA	NA	Yes	Black	0.4	No	13
Kluger and Saarinen, 2015 [31]	M	50	>60	NA (does not remember)	NA	Left arm	No	BCC	Yes	Yes	Dark blue	In situ	No	NA
Anthony et al., 2015 [32]	M	76	>20	NA (does not remember)	≤6 months	Right upper arm	NA	MM and NMSC	NA	Yes	Dark blue	In situ	No	8 × 20
Joyce et al., 2015 [33]	M	33	3	NA (does not remember)	NA	Chest	NA	No	Yes	Yes	Red	11	Positive	NA
Caccavale et al., 2015 [34]	M	49	NA	10	NA	Right upper arm	NA	NA	NA	Yes	Dark blue	0.4	No	NA
Tchernev et al., 2015 [35]	M	42	1	Pre-existing nevus (4 years)	NA	Back	No	No	Yes (sunburns)	Yes	Blue	0.3	No	NA
Deinlen et al., 2016 [36]	F	34	NA	NA	NA	Right lower leg	NA	NA	NA	Yes	Dark blue	1.3	Negative	NA
Armegot-Carbò et al., 2016 [37]	M	35	2	Pre-existing nevus	Change over 2 years	Right arm	NA	NA	NA	Yes	Black	0.35	No	NA
	M	82	60	NA (does not remember)	NA	Left arm	No	No	NA	Yes	Black	0.4	No	NA
Ricci et al., 2018 [38]	M	38	9	Pre-existing nevus (5 years)	1	Left pectoral region	NA	NA	Yes (no sunburns)	Yes	Dark blue	0.4	No	8 × 10
Cherkaoui el Baraka et al., 2019 [39]	M	61	10	1	NA	Back	NA	NA	NA	Yes	Dark blue	7	No	4.5 × 4.5
	M	39	10	NA	0.5	Left arm	NA	NA	Yes	Yes	Black	0.9	No	NA
Veitch et al., 2019 [40]	M	76	≈60	NA (long-standing mole)	NA	Right forearm	NA	MM	NA	Yes	Black	4.9	NA	NA
Ricci et al., 2021 [2]	M	34	10	NA	0.3	Left arm	No	No	Yes	Yes	Black	In situ	No	3 × 4
Leijs et al., 2021 [41]	M	52	NA	NA	NA	Lower back	NA	NA	NA	Yes	Black	0.7	Positive	40 × 60
	M	33	NA	NA (appeared after tattoo)	NA	left shoulder/scapula	NA	NA	NA	NA	NA	6	Positive	NA
Monfrecola et al., 2023 [42]	F	32	11	11	NA	Back	NA	NA	NA	Yes	Black	3	Negative	20 × 30
Vasanthan et al., 2023 [43]	M	59	>10	NA (does not remember)		Chest	NA	NA	NA	Yes	Blue	0.29	No	NA
Fidanzi et al., 2024 [44]	M	39	>30	Pre-existing nevus	NA	Back	No	NA	Yes	Yes	Black	0.3	No	NA
Zuberi et al., 2024 [45]	F	37	5	5	NA	Right forearm	NA	NA	NA	Yes	Black	In situ	No	13 × 12
Our cases, 2024	M	47	10	Pre-existing nevus	Change in appearance over 1 year	Right arm	Yes (maternal aunt)	Two MM: in situ and stage IA	Yes (sunburns)	Yes	Dark blue/green	0.4	No	16 × 18
	M	40	NA	NA	NA	Left upper back	No	MM in situ	Yes	Yes	Black	0.4	No	7 × 9

**Table 2:** Substances in tattoo inks with potential toxic effects [4,5,6,7,8].

Chemical Substance	Potential Risks	Common Uses in Tattoo Ink
Heavy Metals	Skin irritation, organ damage, carcinogenic potential	Used as pigments or contaminants
Aluminum	Skin irritation, potential neurotoxicity	Used in certain pigments
Arsenic	Carcinogenic, toxic	Contaminant in pigments
Barium	Toxicity, including cardiovascular and gastrointestinal effects	Used as a pigment in some inks
Cadmium	Toxic to kidneys and bones	Found in yellow, red, and orange pigments
Chromium	Allergen, carcinogenic in some forms	Found in green inks or as a contaminant
Cobalt	Allergen, toxic in high doses	Found in blue pigments
Lead	Toxic to nervous system and kidneys	Found in red, yellow, and orange inks
Mercury	Neurotoxic	Found in some red pigments
Nickel	Allergen, possible carcinogen	Impurities in pigments, especially metallic inks
Azo Dyes	May release carcinogenic aromatic amines upon degradation	Used for bright and vivid colors
Polycyclic Aromatic Hydrocarbons (PAHs)	Carcinogenic, mutagenic, toxic to reproduction	Found in black inks, often derived from soot or carbon black
Benzo(a)pyrene	Strongly carcinogenic	Found in black inks containing PAHs
Carbon Black	May contain PAHs, lung irritation if inhaled	Found in black inks
Formaldehyde and Formaldehyde-releasing Agents	Allergic reactions, carcinogenic	Preservatives in some inks
Isopropanol (Isopropyl Alcohol)	Skin irritation, dryness, systemic effects at high doses	Solvent and disinfectant in ink formulations
Methanol	Neurotoxicity, blindness, systemic toxicity	Sometimes found as a contaminant in inks
Parabens	Endocrine disruption, allergic reactions	Preservative in some ink formulations
Phthalates	Endocrine disruption, reproductive toxicity	Plasticizers to improve ink fluidity
Titanium Dioxide (TiO_2_)	Skin irritation, potentially harmful when exposed to UV light	Common white pigment, also used to lighten other colors
Zinc Oxide	Skin irritation, potential systemic toxicity	Used in white and some pastel-colored inks

## Data Availability

The raw data supporting the conclusions of this article will be made available by the authors on request.
